# Transcriptional and Hormonal Responses in Ethephon-Induced Promotion of Femaleness in Pumpkin

**DOI:** 10.3389/fpls.2021.715487

**Published:** 2021-09-01

**Authors:** Qingfei Li, Weili Guo, Bihua Chen, Feifei Pan, Helian Yang, Junguo Zhou, Guangyin Wang, Xinzheng Li

**Affiliations:** ^1^College of Horticulture and Landscape, Henan Institute of Science and Technology, Xinxiang, China; ^2^Henan Province Engineering Research Center of Horticultural Plant Resource Utilization and Germplasm Enhancement, Xinxiang, China

**Keywords:** endogenous hormone, ethephon, pumpkin, sex differentiation, yield

## Abstract

The number and proportion of female flowers per plant can directly influence the yield and economic benefits of cucurbit crops. Ethephon is often used to induce female flowers in cucurbits. However, the mechanism through which it affects floral sex differentiation in pumpkin is unknown. We found that the application of ethephon on shoot apical meristem of pumpkin at seedling stage significantly increased the number of female flowers and expedited the appearance of the first female flower. These effects were further investigated by transcriptome and hormone analyses of plants sprayed with ethephon. A total of 647 differentially expressed genes (DEGs) were identified, among which 522 were upregulated and 125 were downregulated. Gene ontology (GO) and Kyoto encyclopedia of genes and genomes (KEGG) analysis indicated that these genes were mainly enriched in plant hormone signal transduction and 1-aminocyclopropane-1-carboxylate oxidase (ACO). The results suggests that ethylene is a trigger for multiple hormone signaling, with approximately 4.2% of the identified DEGs involved in ethylene synthesis and multiple hormone signaling. Moreover, ethephon significantly reduced the levels of jasmonic acid (JA), jasmonoyl-L-isoleucine (JA-ILE), and para-topolin riboside (pTR) but increased the levels of 3-indoleacetamide (IAM). Although the level of 1-aminocyclopropanecarboxylic acid was not changed, the expression of *ACO* genes, which code for the enzyme catalyzing the key rate-limiting step in ethylene production, was significantly upregulated after ethephon treatment. The results indicate that the ethephon affects the transcription of ethylene synthesis and signaling genes, and other hormone signaling genes, especially auxin responsive genes, and modulates the levels of auxin, jasmonic acid, and cytokinin (CK), which may together contribute to femaleness.

## Introduction

Pumpkin (*Cucurbita moschata* Duch.) is cultivated worldwide and is popular for its high nutritional and medicinal value (Lu et al., 2019). As a monoecious plant, pumpkin is a typical material for exploration of floral sex differentiation. The sex differentiation in the flowers of pumpkin includes three stages. First, male flowers appear at the base nodes of the plant, and almost no female flowers are generated at this stage. In the second stage, female flowers alternate with male flowers; usually, one female flower appears after several male flowers. In the third stage, which occurs at the end of the blooming season, female flowers appear continuously, but these are not suitable for generating fruits. Overall, male flowers are much more abundant than female flowers in pumpkin. However, for higher yield, it is necessary to have more female flowers per plant.

Sexual differentiation of flowers is mainly influenced by sex determining genes, hormones, and environmental factors. Sex expression in cucurbitaceae crops can be affected by several phytohormones, such as ethylene, auxin, gibberellin, cytokinin, abscisic acid (ABA), brassinosteroid, and salicylic acid (SA; Byers et al., 1972; Rudich and Halevy, 1974; Trebitsh et al., 1987; Menéndez et al., 2009; Pimenta Lange and Lange, 2016; Zhang et al., 2017). Among these, ethylene is the main regulator of sex determination in cucurbitaceae (Manzano et al., 2011).

Sex differentiation in cucumber, which has been studied more intensively among cucurbitaceae crops, is mainly determined by the *F* (*CsACS1G*), *M* (*CsACS2*), and *A* (*CsACS11*) genes. These genes encode 1-aminocyclopropane-1-carboxylate synthase (ACS), a key rate-limiting enzyme in the biosynthesis of ethylene (Pan et al., 2018). The *F* gene promotes femaleness (Mibus and Tatlioglu, 2004; Knopf and Trebitsh, 2006), the *M* gene inhibits the development of stamens (Yamasaki et al., 2001; Saito et al., 2007), and the *A* gene is an androecious gene (plants with mutations in *CsACS11* do not have female flowers; Boualem et al., 2015). 1-aminocyclopropane-1-carboxylate oxidase (ACO) is another key enzyme in the ethylene biosynthesis pathway (Adams and Yang, 1979; Houben and Van de Poel, 2019). Organ-specific overexpression of cucumber *CsACO_2_* was reported to arrest the development of stamens in *Arabidopsis*. Among floral organs, stamens are the most sensitive to exogenous ethylene, and their development can be arrested by endogenous ethylene for inducing female flowers (Duan et al., 2008). Recently, it was shown that *ACO* is expressed in the carpel primordia and is required for the development of carpel in cucumber. Cucumber plants having a mutation in *CsACO_2_* bear only male flowers because of impaired enzymatic activity of ACO and reduced emission of ethylene. In addition, a transcription factor, *CsWIP1*, which is negatively correlated with the formation of female flowers, could repress the expression of *CsACO_2_* by binding to its promoter (Chen et al., 2016). These findings suggest that *ACO* is indispensable for the development of female flowers.

Besides the ethylene biosynthesis genes (*ACS*, *ACO*), many genes related to ethylene signaling have recently been reported to be involved in sex differentiation. Sex expression is a complex process. In a comparative transcriptome analysis of shoot apices from male, female, and hermaphroditic lines of cucumber, hormone synthesis and signaling, and ion homeostasis, which is important for ethylene perception and signaling, were found to be involved in sex differentiation (Pawełkowicz et al., 2019). *CpETR1A* and *CpETR2B* are ethylene receptor genes in *Cucurbita pepo*, which control the ethylene response; mutants in *CpETR1A* and *CpETR2B* are ethylene-insensitive and exhibit conversion from monoecy to andromonoecy (García et al., 2019). The ethylene-receptor gene, *CsETR1*, expressed in the pistil primordia, is involved in the arrest of stamen development by inducing DNA damage in primordial anthers of female flowers (Hao et al., 2003; Yamasaki et al., 2003; Duan et al., 2008; Wang et al., 2010). The mRNA levels of *Cs-ETR2* and *C_S_-ERS* were significantly enhanced after ethrel application and were decreased upon application of an ethylene inhibitor (Yamasaki et al., 2000). We previously performed a comparative analysis of the transcriptomes of aborted and normal pistils, and showed that ethylene signal transduction genes are implicated in the development of pistils in *C. moschata* (Li et al., 2020).

Flower development involves interaction of multiple hormones. Besides ethylene, auxin is another important regulator of flower development, and the signaling pathways of the two are suggested to crosstalk (Muday et al., 2012). Auxin response factors (ARFs) were shown to be indispensable for the development of pistils in Japanese apricot (Song et al., 2015). *CpARF* is highly expressed during the early stages of flower development. Many auxin response elements (AuxREs) are present in the promoters of ethylene signaling (*CpETR*) and biosynthesis (*CpACS*, *CpACO*) genes (Liu et al., 2015). Ethylene stimulates the biosynthesis of auxin and its transport toward the elongation zone in the root tip; root cell expansion is, therefore, a result of coordinated actions of ethylene and auxin pathways (Vanstraelen and Benková, 2012).

Ethephon is a widely used ethylene-releasing agent in agriculture. When applied to plants, ethephon acts *via* release of ethylene, which can interfere in the growth process of plants (Cooke and Randall, 1968; Sun et al., 2015). In the field production of cucurbits, ethephon is used to induce more female flowers to obtain higher yields. Treatment with 50mg/L ethrel at the third leaf stage has long been known to increase the femaleness of cucumber (Iwahori et al., 1970). The number of female flowers per plant was increased to varying degrees upon treatment with ethrel in the concentration range from 50 to 250mg/L (Jin et al., 2011). As described above, in cucumber, ethylene may arrest the development of stamens and promote the generation of female flowers. In *Ficus carica*, ethephon treatment induced changes at the molecular level; for example, it significantly upregulated *ACO* and *ARF* and downregulated most of the *ERF* and *PAL* genes (Cui et al., 2021). Although ethephon has been used to regulate floral sex differentiation in the production of cucurbits for many years, little is known about the underlying regulatory mechanisms in pumpkin. Whether ethephon affects floral sex differentiation in pumpkin by regulating gene transcription associated with floral development or by modulating the endogenous levels of hormones and which hormones respond to ethephon is unclear. In the present study, the application of ethephon was observed to induce more female flowers in pumpkin and expedited the appearance of the first female flower. To further explore the regulatory mechanism behind the effect of ethephon in sex differentiation, we investigated the changes in the transcriptome and levels of endogenous hormones in the shoot apical meristem after external application of ethephon. Our results indicate that the early flowering and the greater number of female flowers might be the result of increased level of the *ACO* transcript and alteration in the expression of hormone signaling genes and endogenous hormones levels.

## Materials and Methods

### Plant Materials and Sample Preparation

An inbred line of pumpkin (*C. moschata* Duch), 009-1, was used as the plant material. The seeds were sterilized with hot water (55°C) for 10–15min, kept soaked for 4–5h, and germinated using soaked germination paper, in petri dishes at 28°C. When 90% of the seeds were germinated, they were transferred into a mixed matrix (peat:vermiculite:perlite, 3:1:0.5, v/v) in pots (diameter, 34cm) and grown under natural light.

### Ethephon Application, Plant Sampling, and Floral Sex Differentiation

Shoot apical meristems were treated twice with ethephon (100mg/L; E8021, Solarbio, Beijing, China) containing 0.1% Tween-20, every 2days, when the third true leaf of seedlings unfolded. Tween-20 (0.1%) was used as a blank control. The concentration of ethephon was chosen according to Yang et al. (2015). After the first exposure to ethephon for 4h, the shoot apical meristems were immediately frozen in liquid nitrogen and stored at −80°C for RNA-sequencing (RNA-seq) and quantitative real-time PCR (qRT-PCR) validation. The results of a preliminary experiment showed no significant effect on gene expression within 4h of ethephon treatment. Hormone levels were analyzed a day after the second treatment. The shoot apical meristems from three plants were pooled as one biological replicate for both ethephon and control treatments. RNA-seq analysis and hormone quantification were performed using three biological replicates. The tissue samples were immediately frozen in liquid nitrogen and then stored at −80°C.

At the flowering stage, the number of female and male flowers within 20 nodes and the node at which the first female flower occurred were determined. The nodes of plants were marked with a red line after every observation, to prevent missing or duplicating the count of flowers. Each group had three plants, each with three biological replicates.

### RNA Sequencing and Analysis

RNA was isolated from shoot apices subjected to ethephon and control treatments using the TRIzol™ reagent (Invitrogen, Carlsbad, CA, United States). The concentration of RNA samples was measured using NanoDrop DU8000 (Thermo, CA, United States), and their purity and integrity were evaluated by agarose gel electrophoresis and using the Agilent 2100 system (Agilent Technologies, CA, United States). Qualified RNA samples were used for construction of cDNA libraries as described previously (Li et al., 2020). The high-quality libraries were used for paired-end sequencing (2×150bp) on the Illumina NovaSeq 6000 System.

The raw reads were processed to filter out the adaptor sequences and low-quality reads (more than 50% bases with SQ≤20 in one read and with more than 10% N bases). The Q20, Q30, GC-content, and sequence duplication level of the clean data were calculated. The clean reads were then mapped to the *C. moschata* genome by using TopHat2 (Trapnell et al., 2012; Kim et al., 2013; Sun et al., 2017), allowing up to one mismatch. The DESeq R package (1.10.1) was used to identify the differentially expressed genes (DEGs) (Love et al., 2014). The fragments per kilobase of exon per million fragments mapped (FPKM) method was used to estimate the expression levels of genes. Values of *p* were adjusted using the Benjamini and Hochberg’s method for controlling the false discovery rate (FDR; Benjamini and Hochberg, 1995). Genes with an adjusted value of *p*<0.05 and a fold change ≥1.5 based on three biological replicates were considered differentially expressed.

Functional annotation of genes was based on the following databases: the NCBI non-redundant (Nr) protein sequences, Swiss–Prot, clusters of orthologous groups of proteins (KOG), protein family (Pfam), gene ontology (GO), and the Kyoto encyclopedia of genes and genomes (KEGG).

### Functional Enrichment Analysis

Gene ontology enrichment analysis of DEGs was implemented using the GOSeq R package (Young et al., 2010), in which gene length bias was corrected, and a *p* value of DEGs ≤0.05 was considered as significantly enriched. KEGG pathway enrichment analysis of DEGs was performed using the software KOBAS (Mao et al., 2005). Pathways with their Benjamini and Hochberg adjusted values of *p*≤0.05 were defined as significantly enriched by DEGs.

### Quantitative Real-Time RT-PCR

The first-strand cDNA was obtained using the PrimeScript™ RT Master Mix (Perfect Real Time) Reagent Kit (Takara, Dalian, China). The qRT-PCR was carried out using a Bio-Rad IQ5 instrument (Foster City, CA, United States), as follows: 95°C for 40s and 40cycles of 95°C for 5s and 61°C for 30s. *ACTIN* was used as an internal control. The primers used for qRT-PCR are listed in Supplementary Material. The expression levels were calculated using the 2^-ΔΔCt^ method (Livak and Schmittgen, 2001). The expression of each gene was determined using three biological and three technical replicates. Furthermore, the correlation analysis and the Pearson correlation coefficient between the log2 (fold change) values obtained in qRT-PCR and RNA-seq were calculated using the IBM SPSS statistics 22 software.

### Quantification of Hormones

The quantification of endogenous auxin, abscisic acid (ABA), jasmonic acid (JA), cytokinin (CK), gibberellic acid (GA), salicylic acid (SA), and 1-aminocyclopropane 1-carboxylic acid (ACC) was performed. Fresh shoot apical meristems (50mg) were frozen in liquid nitrogen and extracted with 1ml methanol/water/formic acid (15:4:1, V/V/V). The extracts were evaporated to dryness under nitrogen gas, reconstituted in 100μl 80% (V/V) methanol, and filtered through a 0.22μm filter for LC-MS analysis. The extracts were analyzed using a UPLC-ESI-MS/MS system (UPLC, ExionLC™ AD, MS, Applied Biosystems 6500 Triple Quadrupole). The analytical conditions were as follows: for LC: column, Waters ACQUITY UPLC HSS T3 C18 (1.8μm, 100mm×2.1mm i.d.); solvent system, water with 0.04% acetic acid (A), acetonitrile with 0.04% acetic acid (B); flow rate, 0.35ml/min; temperature, 40°C; injection volume, 2μl; for MS/MS: AB 6500+ QTRAP® LC–MS/MS System, equipped with an ESI Turbo Ion-Spray interface, operating in both positive and negative ion modes and controlled using the Analyst 1.6 software (AB Sciex). Solutions containing different concentrations (0.01, 0.05, 0.1, 0.5, 1, 5, 10, 50, 100, 200, and 500ng/ml) of 1-aminocyclopropanecarboxylic acid, indole-3-acetyl-L-aspartic acid, 3-indoleacetamide (IAM), para-topolin riboside (pTR), jasmonic acid, jasmonoyl-L-isoleucine (JA-ILE), cis(+)-12-oxophytodienoic acid, and gibberellin A15 were used to generate standard curves for each hormone.

## Results

### Effect of Ethephon on Floral Sex Differentiation in Pumpkin

**Figure 1:**
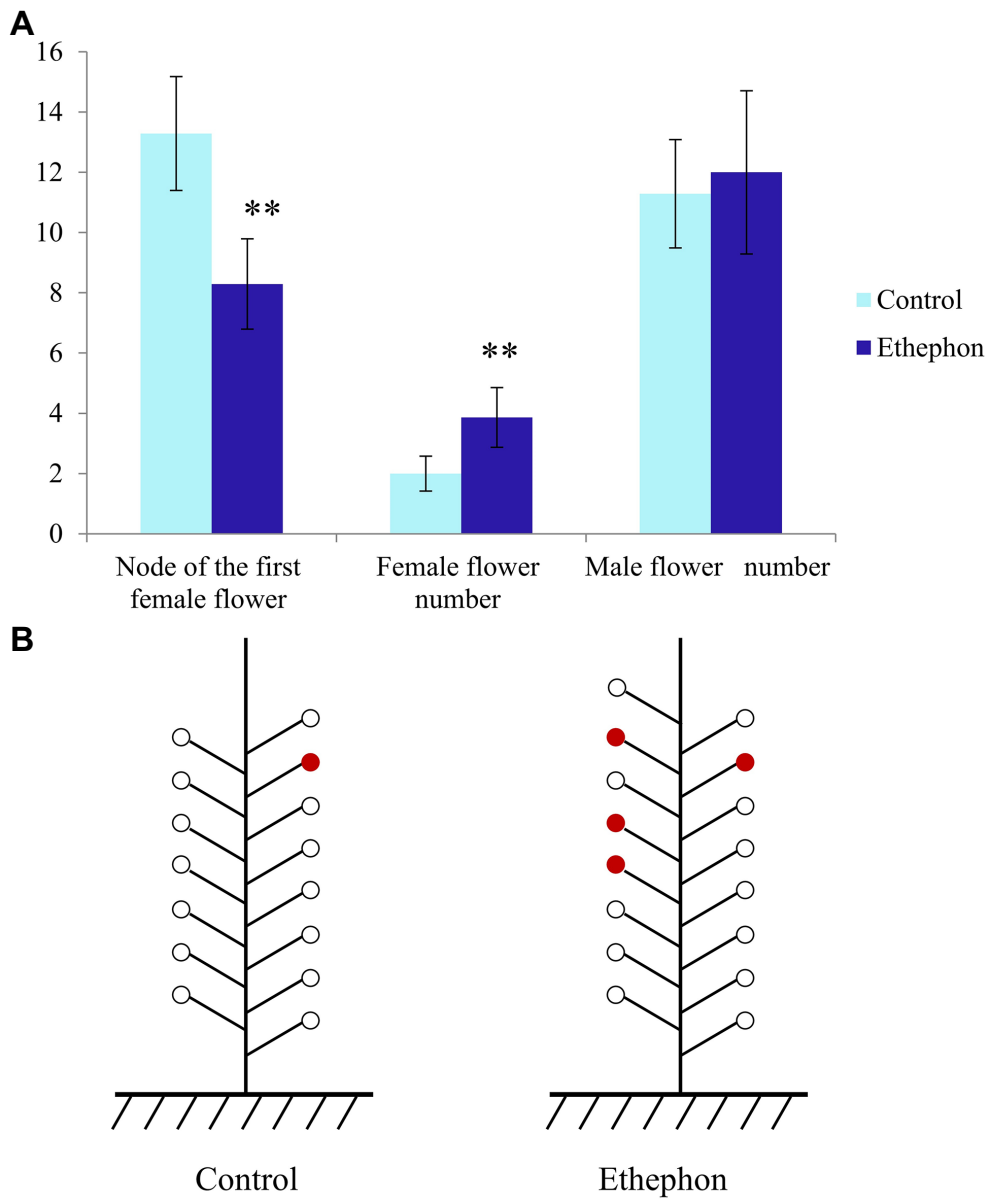
Effect of ethephon on floral sex differentiation of pumpkin. **(A)** Node of the first female flower and the number of female flower and male flower in ethephon-treated and control plants. Double asterisk indicates a significant difference between control and ethephon-treated plants at 1% level as determined by Duncan’s new multiple range test. **(B)** The schematic diagram of sex differentiation of ethephon-treated and control plants.

The floral sex differentiation in pumpkin plants treated with ethephon was investigated. Ethephon treatment significantly expedited the appearance of the first female flower from node 13.29±1.89 (in the control) to node 8.29±1.50. The number of female flowers within 20 nodes in ethephon-treated plants was significantly higher (3.86±0.99) than in the control (2.00±0.58). The effect of ethephon treatment on the number of male flowers was not obvious (Figures 1A,B).

### Statistical Analysis of RNA-seq Data and DEGs Induced by Ethephon

**Figure 2:**
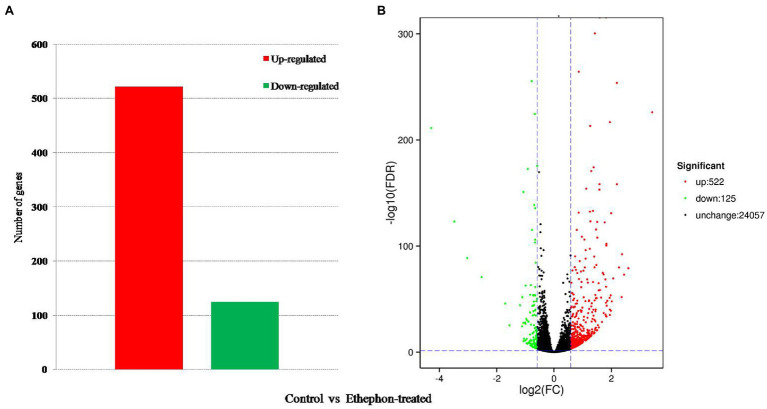
Analysis of differentially expressed genes (DEGs) between ethephon-treated and control plants. **(A)** Numbers of upregulated and downregulated DEGs. Red and green colors indicate up- and downregulated transcripts, respectively. **(B)** Volcano map of DEGs.

An average of 45,929,596 reads was obtained in the sequencing of the cDNA library prepared from each sample, with an average Q30 quality score≥93.30% and average Q20 quality score≥97.52%. The alignment of filtered reads with the *C. moschata* genome sequence revealed an average mapping percentage of 95.06% (Table 1). A total of 647 DEGs, including 522 upregulated and 125 downregulated genes, were identified by comparing the transcriptomes of the shoot apical meristem of ethephon-treated and control plants (Figure 2).

**Table 1:** Statistical analysis of reads mapped to the reference genome after rRNA filtering.

Samples	Total reads	Q20 (%)	Q30 (%)	Mapped reads	Mapping rate (%)
C1	49,013,376	97.68	93.60	46,836,934	95.56
C2	43,642,098	97.43	93.10	41,485,719	95.06
C3	47,105,588	97.46	93.20	44,747,589	94.99
EY1	46,217,576	97.48	93.22	43,778,467	94.72
EY2	47,759,520	97.48	93.22	45,242,692	94.73
EY3	41,839,420	97.59	93.45	39,865,629	95.28
Average	45,929,596	97.52	93.30	43,659,505	95.06

*C1, C2, and C3 represent the three biological replicates of the control; EY1, EY2, and EY3 represent the three biological replicates of ethephon treatment*.

### Functional Enrichment of DEGs

**Figure 3:**
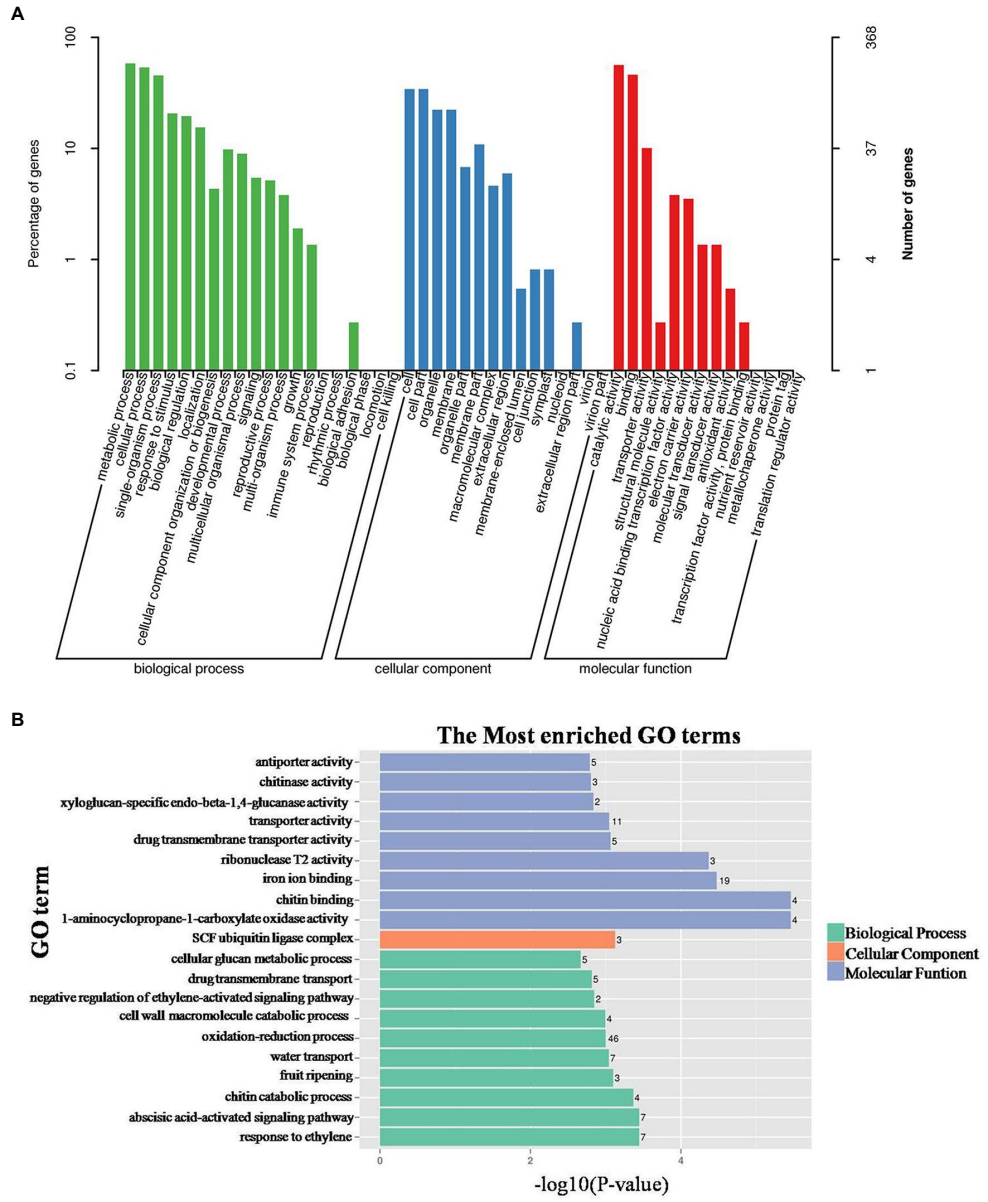
Gene ontology (GO) analysis of DEGs between control and ethephon-treated shoot apical meristem of pumpkin. **(A)** GO categories. **(B)** Enrichment analysis of DEGs in biological processes, cellular components, and molecular functions category. The number of DEGs enriched in each term is marked on the left.

Gene ontology analysis indicated that the annotated genes were enriched in three major functional categories: biological processes, cellular components, and molecular functions. In the biological process category, most of the transcripts were enriched in metabolic processes, cellular process, and single-organism process (Figure 3A). In this category, the most significantly enriched GO terms included response to ethylene (GO:0009723), abscisic acid-activated signaling pathway (GO:0009738), and chitin catabolic process (GO:0006032; Figure 3B). In the cellular component category, DEGs were mainly enriched in cell, cell part, organelle, and membrane (Figure 3A), among which the SCF ubiquitin ligase complex (GO:0019005) was the most significantly enriched GO term (Figure 3B). In the molecular function category, catalytic activity and binding were highly enriched GO terms (Figure 3A), and the most significantly enriched GO terms were ACO and chitin binding (Figure 3B). More DEGs were enriched in the biological processes and cellular components categories, and relatively fewer DEGs were enriched in the molecular functions category.

**Figure 4:**
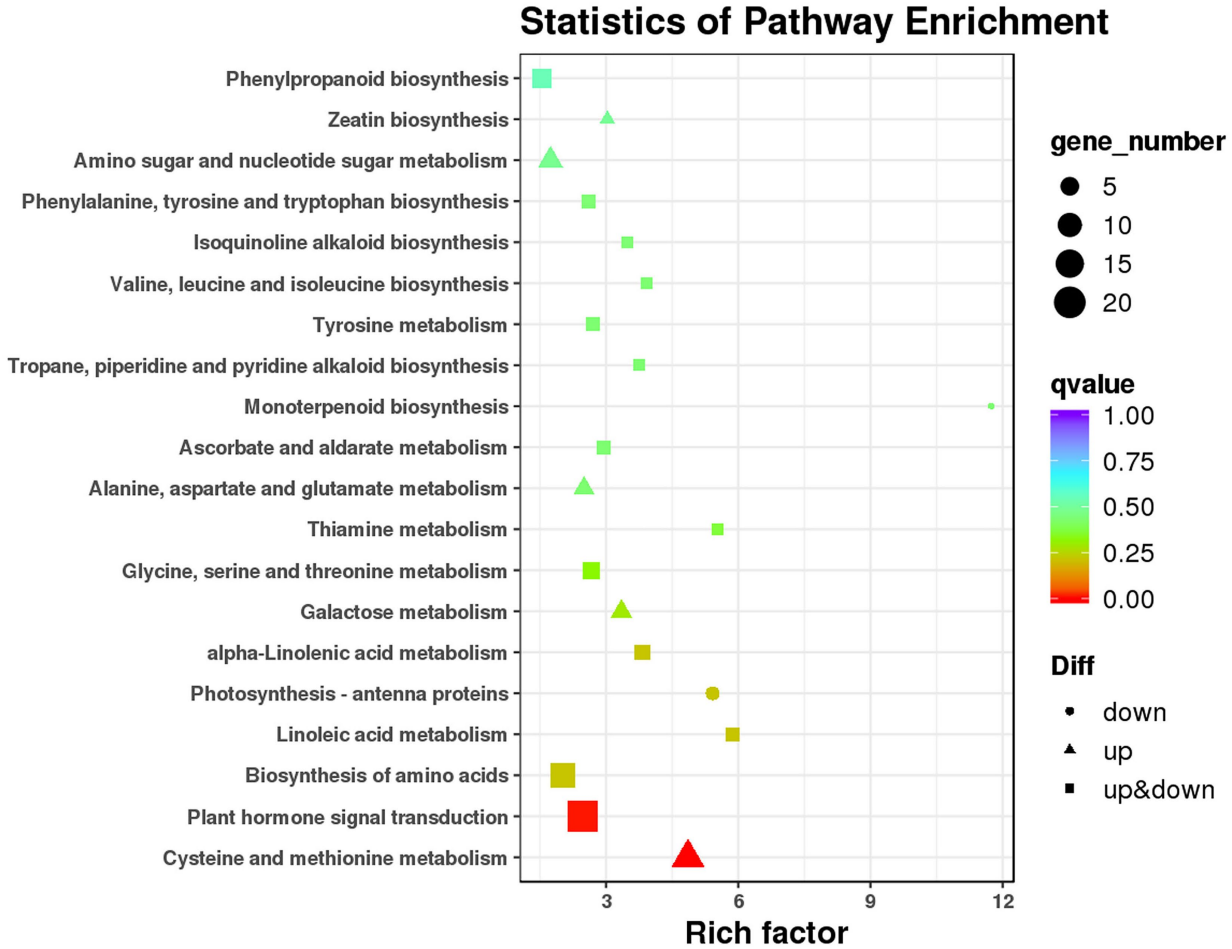
Kyoto encyclopedia of genes and genomes (KEGG) enrichment analysis of DEGs.

**Figure 5:**
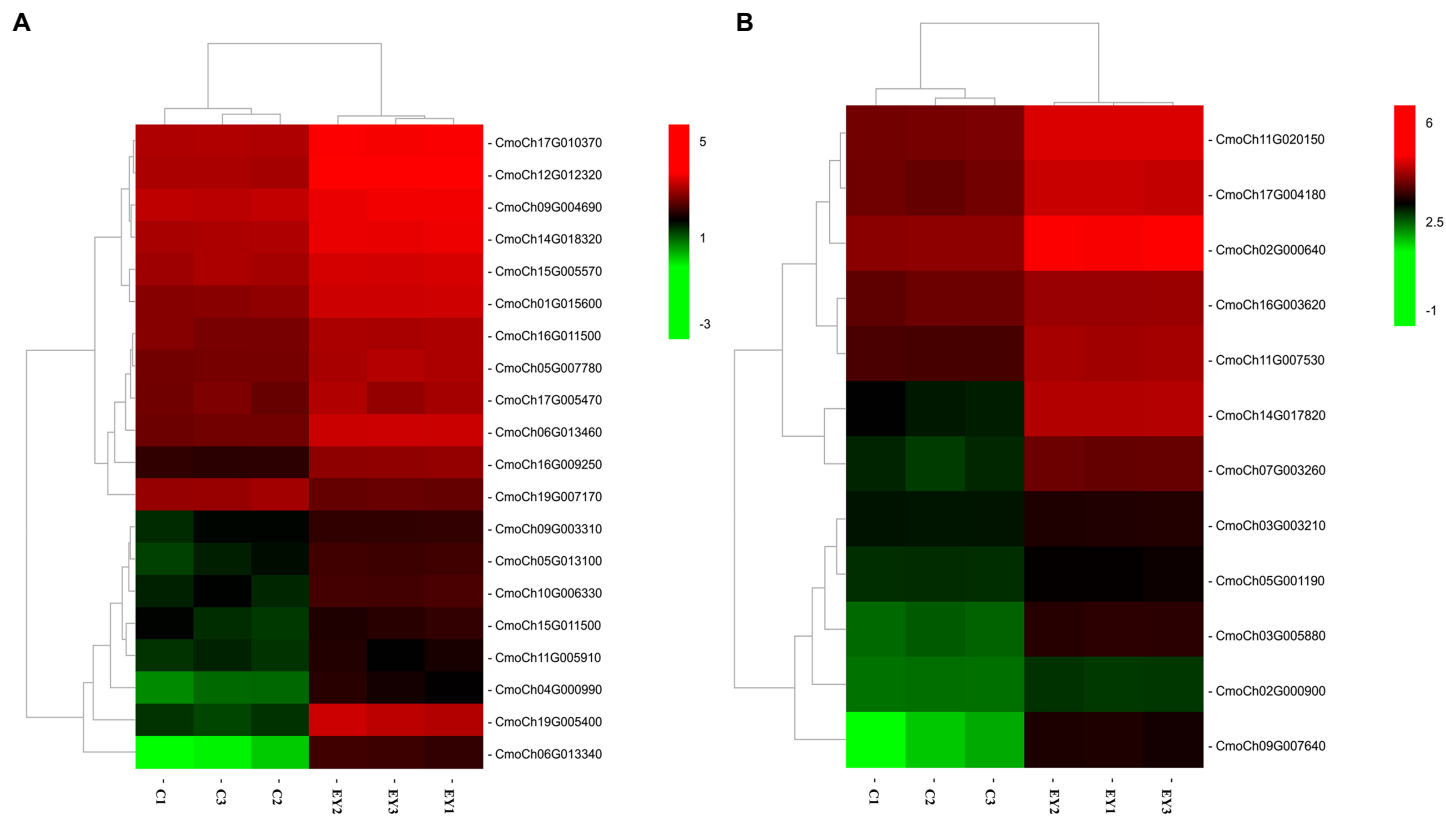
Heatmap diagram of expression levels of DEGs in significant enrichment KEGG pathways. **(A)** DEGs enriched in plant hormone signal transduction pathway. **(B)** DEGs enriched in cysteine and methionine metabolism pathway. The red and green colors indicate high and low expression levels (log^2^ FPKM), respectively.

Kyoto encyclopedia of genes and genomes pathway analysis was performed to investigate the pathways that responded to ethephon treatment. A total of 116 DEGs were mapped to 72 KEGG pathways. The top 20 pathways in KEGG enrichment analysis are as shown in Figure 4. Among them, DEGs were significantly enriched in cysteine and methionine metabolism pathways and plant hormone signal transduction pathway (Figure 5). Notably, all the 12 DEGs enriched in the cysteine and methionine metabolism pathways were upregulated. Five of them were annotated as predicted *ACO* genes (Table 2). Twenty DEGs, including 19 upregulated and one downregulated genes, were enriched in plant hormone signal transduction pathway. Among them, 10 DEGs were involved in ethylene response and four were involved in auxin response and induction, according to the annotations in the Nr and Swiss-Prot databases (Table 2). Except for one auxin-induced gene, all other DEGs significantly enriched in the plant hormone signal transduction and cysteine and methionine metabolism pathways were upregulated upon ethephon treatment.

**Table 2:** List of DEGs enriched in plant hormone signal transduction pathway (No. 1–20) and cysteine and methionine metabolism pathway (No. 21–32).

Number	Gene ID	Gene annotation	log_2_FC	Regulated
1	CmoCh01G015600	EIN3-binding F-box protein 1	0.9421233	up
2	CmoCh04G000990	Two-component response regulator ARR17	0.9411019	up
3	CmoCh05G007780	Ethylene receptor 1 GN=ETR1	0.7827862	up
4	CmoCh05G013100	EIN3-binding F-box protein 1	1.168263	up
5	CmoCh06G013340	Ethylene-responsive transcription factor 1B	1.9466826	up
6	CmoCh06G013460	Ethylene receptor 2 GN=ETR2	1.300988	up
7	CmoCh09G003310	Abscisic acid (ABA) receptor PYL2	0.7471918	up
8	CmoCh09G004690	EIN3-binding F-box protein 1	0.702505	up
9	CmoCh10G006330	Auxin-responsive protein IAA11	0.990624	up
10	CmoCh11G005910	Auxin-induced protein AUX22	0.608669	up
11	CmoCh12G012320	EIN3-binding F-box protein 1	1.262806	up
12	CmoCh14G018320	Ethylene receptor 2 GN=ETR2	0.9009867	up
13	CmoCh15G005570	EIN3-like 1 protein	0.6573002	up
14	CmoCh15G011500	Auxin-induced protein 22D	0.6962115	up
15	CmoCh16G009250	Indole-3-acetic acid-amido synthetase GH3 auxin-responsive promoter	1.3620471	up
16	CmoCh16G011500	Serine/threonine-protein kinase SAPK3	0.587906	up
17	CmoCh17G005470	Abscisic acid receptor PYL9	0.6050516	up
18	CmoCh17G010370	Ethylene receptor GN=ETR1	1.0596167	up
19	CmoCh19G005400	Basic form of pathogenesis-related protein 1	2.5933011	up
20	CmoCh19G007170	Auxin-induced protein 15A	−0.635074	down
21	CmoCh02G000640	1-aminocyclopropane-1-carboxylate oxidase 1	1.5946206	up
22	CmoCh02G000900	Tyrosine aminotransferase GN=TAT	0.7062581	up
23	CmoCh03G003210	Alanine–glyoxylate aminotransferase 2 homolog 3	0.6897043	up
24	CmoCh03G005880	1-aminocyclopropane-1-carboxylate oxidase 3	1.7959392	up
25	CmoCh05G001190	Branched-chain-amino-acid aminotransferase 2	0.7311296	up
26	CmoCh07G003260	1-aminocyclopropane-1-carboxylate oxidase 3	1.9930183	up
27	CmoCh09G007640	1-aminocyclopropane-1-carboxylate oxidase 5	2.4427595	up
28	CmoCh11G007530	Methylthioribose-1-phosphate isomerase	1.2713346	up
29	CmoCh11G020150	1-aminocyclopropane-1-carboxylate oxidase 1	1.4275079	up
30	CmoCh14G017820	1,2-dihydroxy-3-keto-5-methylthiopentene dioxygenase	2.3726478	up
31	CmoCh16G003620	Methylthioribose kinase	0.6691512	up
32	CmoCh17G004180	L-3-cyanoalanine synthase 1	1.2548785	up

### Validation of RNA-Seq Results by qRT-PCR

**Figure 6:**
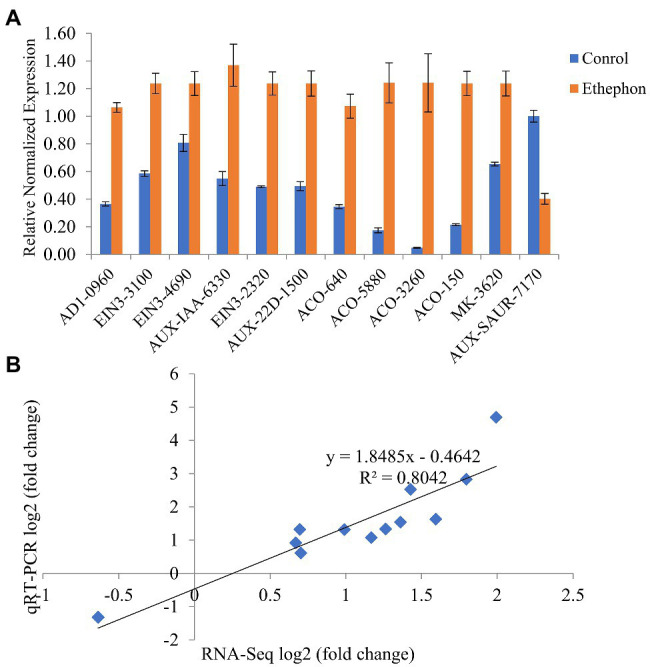
Quantitative real-time PCR (qRT-PCR) verification. **(A)** Relative expression levels of 12 selected DEGs between ethephon-treated and control plants. Error bars indicate the SEs. **(B)** Correlation analysis of RNA-seq log2 (fold change) and qRT-PCR log2 (fold change) of 12 selected genes: AD1-0960 (CmoCh09G010960.1), ETHYLENE INSENSITIVE 3 (EIN3)-3100 (CmoCh05G013100.1), EIN3-4690 (CmoCh09G004690.1), AUX-IAA-6330 (CmoCh10G006330.1), EIN3-2320 (CmoCh12G012320.1), AUX-22D-1500 (CmoCh15G011500.1), AUX-SAUR-7170 (CmoCh19G007170.1), ACO-640 (CmoCh02G000640.1), ACO-5880 (CmoCh03G005880.1), ACO-3260 (CmoCh07G003260.1), ACO-150 (CmoCh11G020150.1), and MK-3620 (CmoCh16G003620.1).

The validation of the transcriptome data was done by qRT-PCR analysis. Twelve DEGs were chosen for validation. The expression levels of these genes determined by qRT-PCR were in good agreement with the RNA-seq data, with relative coefficient, *R^2^*=0.804 (Figures 6A,B), and the Pearson correlation coefficient, *R*=0.897 (*p*<0.0001). These results indicate the reliability of the RNA-seq analysis performed in this study.

### Changes in the Endogenous Levels of Hormones Upon Ethephon Treatment

The endogenous levels of hormones, *viz*., auxin, ABA, JA, CK, GA, SA, and ACC, in the shoot apical meristem of pumpkin plants subjected to ethephon and control treatments were compared to assess the effects of ethephon. A fold change≥2 was considered to indicate differential hormone levels (Table 3). Ethephon treatment decreased the levels of JA, JA-ILE, and pTR and significantly increased the levels of 3-IAM (Table 3). In addition, the levels of cis(+)-12-oxophytodienoic acid, gibberellin A15, and indole-3-acetyl-L-aspartic acid were slightly decreased, while those of 1-aminocyclopropanecarboxylic acid were not effected by the treatment.

**Table 3:** Phytohormone levels in the shoot apical meristem of control and ethephon-treated plants.

	JA	JA-ILE	IAM	pTR
Control	2.23±0.42^a^	0.47±0.07^a^	1.18±0.23^a^	0.98±0.13^a^
Ethephon	1.05±0.17^b^	0.18±0.05^b^	2.63±0.54^b^	0.36±0.04^b^
Category	JA	JA	Auxin	CK

*Data are means (ng/g) of three replicates±SD. Different letters indicate a significant difference between each hormone level of control plants and that of ethephon-treated plants at 5% level as determined by Duncan’s new multiple range test. JA, jasmonic acid; JA-ILE, jasmonoyl-L-isoleucine; IAM, 3-indoleacetamide; pTR, para-topolin riboside; and CK, cytokinin*.

## Discussion

Ethephon is a plant growth regulator that is mainly used in field production for regulating floral sex differentiation (Papadopoulou et al., 2005; Martínez et al., 2013), promoting fruit ripening (Cui et al., 2021), and breaking dormancy (Corbineau et al., 2014). The effects of ethephon on the sex differentiation phenotype of flowers in zucchini, cucumber, and watermelon are well-known, but the underlying mechanism remains unclear. Moreover, there has been no systematic research on the effect of ethephon on the floral sex expression of pumpkin. In the present study, 100mg/l ethephon was used to treat shoot apical meristem of pumpkin at the seedling stage. The ethephon treatment significantly advanced the appearance of the first female flowers and significantly increased the number of female flowers within 20 nodes. To investigate the mechanism underlying these effects, the changes in the transcriptome and hormone levels upon ethephon treatment were analyzed.

Transcriptome analysis indicated an upregulation of seven DEGs annotated as *ACO*, which encode a key enzyme in ethylene biosynthesis. Study have found that *CsACO_2_* mutants of cucumber bear only male flowers because of impairment of the enzymatic activity of ACO and reduced emission of ethylene (Chen et al., 2016). ACO can sometimes be rate limiting in ethylene biosynthesis (Houben and Van de Poel, 2019). Overexpression of *ACO* from *Vitis vinifera* in tomato was reported to increase the rate of ethylene release (Cai et al., 2013). The results of the above studies indicate that *ACO* is indispensable for the stable emission of ethylene and the development of female flowers. The upregulation of *ACO* in ethephon-treated plants mean that they may have a greater capacity to produce more ethylene.

Ethylene response, including ethylene receptor, ethylene insensitive 3, and ethylene-responsive transcription factor, was found to be the most significantly enriched GO term (Figure 3B). Ethephon treatment upregulated the expression of ethylene response-related genes, which might be due to the increased release of ethylene. ETR controls the ethylene response, and mutations in *CpETR1A* and *CpETR2B* result in ethylene-insensitivity and conversion of monoecy into andromonoecy (García et al., 2019). In cucumber, *CsETR1* is localized in the pistil primordia and is involved in arresting the development of stamen in female flowers (Hao et al., 2003; Yamasaki et al., 2003; Duan et al., 2008; Wang et al., 2010). The application of ethephon increased the transcript levels of ethylene receptor, ethylene-responsive transcription factor, ETHYLENE INSENSITIVE 3 (EIN3)-binding F-box protein 1, auxin-responsive, indole-3-acetic acid-amido synthetase, and abscisic acid receptor genes. These results for pumpkin are in agreement with those of a previous study in which *ACO*s and IAA-amino acid hydrolase, indole-3-acetic acid-amido synthetase, auxin-responsive protein, and ABA receptor genes were found to be upregulated upon ethephon treatment (Cui et al., 2021).

Ethylene treatment induces a cascade of regulatory events. In the present study, we observed that five of the 20 DEGs included in plant hormone signal transduction pathways were annotated as EIN3-binding F-box protein and EIN3-like 1 protein, which were upregulated upon ethephon treatment. EIN3 and EIN3-LIKE1 (EIL1) are key transcription factors for ethylene signaling. EIN3 accumulates in the nucleus in the presence of ethylene. In contrast, in the absence of ethylene, EIN3 is negatively regulated and constantly degraded in plant cells (Cho and Yoo, 2015). The complex regulation of the activation of EIN3 and EIL1 in response to ethylene involves triggering of primary transcription through EIN3-binding sites in the promoters of *ETHYLENE RESPONSE FACTOR1 (ERF1)*, *EBF2*, *ERS1*, *ERS2*, and *ETR2*, that finally activates the ethylene biosynthesis genes (*ACOs* and *ACSes*) Vandenbussche et al., 2012; Cho and Yoo, 2015; Dolgikh et al., 2019). The upregulation of genes coding EIN3-binding F-box protein and EIN3-like 1 protein may contribute to the changes of transcriptional levels of *ETR* and *ACO* genes in ethephon-treated plants. Besides the classical mechanism of ethylene-induced stabilization of EIN3/EIL1, JA can release EIN3/EIL1 from repression by accelerating the degradation of JAZ, thereby inducing ethylene responses (Zhu et al., 2011). In our study, the levels of JA and JA-ILE were significantly decreased; however, whether this is related to the upregulation of EIN3/EIL1 needs to be investigated.

Auxin and ethylene act synergistically to regulate the growth and development of plants. An increase in the levels of ethylene was reported to elevate the auxin response, monitored using auxin-inducible reporters, in the root elongation zone (Muday et al., 2012). Ethylene may positively regulate auxin synthesis. The levels of free IAA were reported to increase in the root tip upon treatment with ACC (100mM; Růžička et al., 2007). We observed that the levels of the auxin-responsive, indole-3-acetic acid-amido synthetase transcript, and IAM were increased upon ethephon treatment. These results support the notion that ethylene enhances the transport of auxin in the elongation zone, which leads to elevated IAA levels (Muday et al., 2012).

In summary, ethephon treatment enhances the synthesis of ethylene by increasing the expression of *ACO* genes, and promotes ethylene signaling, which may further crosstalks with an upregulation of auxin responsive genes and the increase of auxin levels. The regulatory network of ethephon in female flowers of pumpkins is fairly complex, which interferes transcriptional and hormonal levels, and in this process ethylene and EIN3 play the intermediate pivotal role. This study have confirmed the effects of ethephon on floral sex differentiation in pumpkin and have presented the mechanism through which ethephon promotes femaleness. Further genetic and biochemical analysis would help in clarifying the regulatory mechanism of ethephon on floral sex differentiation.

## Data Availability Statement

The raw transcriptome data have been deposited in the National Center for Biotechnology Information (NCBI) SRA database (accession number: PRJNA736171).

## Author Contributions

QL conducted the experiments and data analysis. WG, BC, FP, and HY assisted with plant material preparation. XL and QL conceived the project. QL wrote the original manuscript. JZ, GW, and XL supervised the studies and reviewed the manuscript. All authors contributed to the article and approved the submitted version.

## Conflict of Interest

The authors declare that the research was conducted in the absence of any commercial or financial relationships that could be construed as a potential conflict of interest.

## Publisher’s Note

All claims expressed in this article are solely those of the authors and do not necessarily represent those of their affiliated organizations, or those of the publisher, the editors and the reviewers. Any product that may be evaluated in this article, or claim that may be made by its manufacturer, is not guaranteed or endorsed by the publisher.
