# Supplementary material for: Transcriptional and Hormonal Responses in Ethephon-Induced Promotion of Femaleness in Pumpkin
Source: Front Plant Sci. 2021 Sep 1;12:715487. doi: 10.3389/fpls.2021.715487 (PMC8442687; doi:10.3389/fpls.2021.715487)
Supplement: Supplementary file 1 [file Data_Sheet_1.PDF]

Supplementary Material

Transcriptional and hormonal responses in ethephon-induced promotion of femaleness in pumpkin

Qingfei Li[1,2], Weili Guo[1,2], Bihua Chen[1,2], Feifei Pan[1,2], Helian Yang[1,2], Junguo Zhou[1,2], Guangyin Wang[1,2], Xinzheng Li[1,2*]

[1] College of Horticulture and Landscape, Henan Institute of Science and Technology, Xinxiang, China;
[2] Henan Province Engineering Research Center of Horticultural Plant Resource Utilization and Germplasm Enhancement, Xinxiang, China

[*] **Correspondence:**
Xinzheng Li
College of Horticulture and Landscape, Henan Institute of Science and Technology
Xinxiang, Henan,453003, China
Email address: lxz2283@126.com

Supplementary Table1. Primers used in this study.

Primer	Primer Sequence（5'-3'）
ACTIN-F	AGCCATCTCTCATCGGTAT
ACTIN-R	CATGGTTGAACCACCACTG
AD1-0960-F	ATGTCTAGCACTGCTGG
AD1-0960-R	TCAGTCTCCCATGCG
EIN3-3100-F	CCTTGTCGGGTTGTTCC
EIN3-3100-R	CCTCTGTTGAATGCTGCTA
EIN3-4690-F	TGTAAGGTCGCTTGAGTG
EIN3-4690-R	ATAGCAGTTGCGGATGG
EIN3-2320-F	GCCGAGGGATGACCGATGT
EIN3-2320-R	ACCGCCGATGCCCAATA
AUX-IAA-6330-F	TCGCAACGAGCCAGAAC
AUX-IAA-6330-R	GCCGCAACTTTGACATAC
AUX-22D-1500-F	CTCGATTTCGTCCACTA
AUX-22D-1500-R	GCTCCCTACTTCCTTGT
AUX-SAUR-7170-F	CTTAGGCCGGAAGCAAGA
AUX-SAUR-7170-R	GGGTGAGTCAAGAGGGAAAT
ACO-640-F	CGAAACAGCACTACAAGAAA
ACO-640-R	CTCGCAAAGTAAATCCAAA
ACO-5880-F	TCAAAGAAATGGTGGCT

ACO-5880-R	TGTTGGAACTGGGAAGAT
ACO-3260-F<br>*ACO-3260-R*	CTGATTGGGAAAGCACA<br>TTAGGACAGGGAGGGTAG
ACO-150-F<br>*ACO-150-R*	TCATCCTCCTCTTCCAG<br>CTAACGACATTCGACCAC
MK-3620-F<br>*MK-3620-R*	ACTGTTCCTTCGCTGTCT<br>AATGCCACTGCCTCAAA
